# Neural Population Dynamics during Reaching Are Better Explained by a Dynamical System than Representational Tuning

**DOI:** 10.1371/journal.pcbi.1005175

**Published:** 2016-11-04

**Authors:** Jonathan A. Michaels, Benjamin Dann, Hansjörg Scherberger

**Affiliations:** 1 German Primate Center, Göttingen, Germany; 2 Faculty of Biology, Georg-August-Universität Göttingen, Göttingen, Germany; Carnegie Mellon University, UNITED STATES

## Abstract

Recent models of movement generation in motor cortex have sought to explain neural activity not as a function of movement parameters, known as representational models, but as a dynamical system acting at the level of the population. Despite evidence supporting this framework, the evaluation of representational models and their integration with dynamical systems is incomplete in the literature. Using a representational velocity-tuning based simulation of center-out reaching, we show that incorporating variable latency offsets between neural activity and kinematics is sufficient to generate rotational dynamics at the level of neural populations, a phenomenon observed in motor cortex. However, we developed a covariance-matched permutation test (CMPT) that reassigns neural data between task conditions independently for each neuron while maintaining overall neuron-to-neuron relationships, revealing that rotations based on the representational model did not uniquely depend on the underlying condition structure. In contrast, rotations based on either a dynamical model or motor cortex data depend on this relationship, providing evidence that the dynamical model more readily explains motor cortex activity. Importantly, implementing a recurrent neural network we demonstrate that both representational tuning properties and rotational dynamics emerge, providing evidence that a dynamical system can reproduce previous findings of representational tuning. Finally, using motor cortex data in combination with the CMPT, we show that results based on small numbers of neurons or conditions should be interpreted cautiously, potentially informing future experimental design. Together, our findings reinforce the view that representational models lack the explanatory power to describe complex aspects of single neuron and population level activity.

## Author Summary

The question of how the brain generates movement has been extensively studied, yet multiple competing models exist. Representational approaches relate the activity of single neurons to movement parameters such as velocity and position, approaches useful for the decoding of movement intentions, while the dynamical systems approach predicts that neural activity should evolve in a predictable way based on population activity. Existing representational models cannot reproduce the recent finding in monkeys that predictable rotational patterns underlie motor cortex activity during reach initiation, a finding predicted by a dynamical model in which muscle activity is a direct combination of neural population rotations. However, previous simulations did not consider an essential aspect of representational models: variable time offsets between neurons and kinematics. Whereas these offsets reveal rotational patterns in the model, these rotations are statistically different from those observed in the brain and predicted by a dynamical model. Importantly, a simple recurrent neural network model also showed rotational patterns statistically similar to those observed in the brain, supporting the idea that dynamical systems-based approaches may provide a powerful explanation of motor cortex function.

## Introduction

Throughout the history of neuroscience research, the question of how motor cortex generates movements has been investigated deeply [1]. Yet, substantial and conflicting models have been proposed [2–7]. According to the representational view, motor cortex neurons encode abstract or high-level aspects of movements, such as kinematic parameters [8]. In contrast, in the dynamical systems view the firing of each neuron is a function of a population optimized to control muscles directly [9]. It remains a point of considerable debate which model better explains existing neural data and provides a mechanistic explanation of how movements can be generated.

The representational view of neuron tuning, or ‘neuron doctrine’, is strongly rooted in the history of neuroscience [10] and detailed models of single neuron tuning have been indispensable tools for a basic understanding of the brain’s computations [11–13]. However, recent advances in electrophysiological recording technology [14,15] have made it possible to examine network level hypotheses of movement generation that require large populations of neurons to study [16–19].

Recently, it was suggested that motor cortex, operating as a dynamical system, might be sufficient for generating required muscle activity [20–22]. Using simultaneous recordings in the dorsal premotor cortex (PMd) and primary motor cortex (M1) of non-human primates, Churchland et al. [22] proposed that preparatory activity may act to prepare a dynamical system, which, like a spring box, could be released to act as an ‘engine of movement’ and produce muscle activity from a basis set of oscillators, which they termed the generator model or dynamical model [9,23]. They supported their theory by developing a dimensionality reduction method (jPCA), which revealed that predictable rotational dynamics underlie a large portion of the variance observed in PMd/M1 during reach initiation, a direct prediction of the dynamical model. Importantly, they showed that representational models of movement activity, including those based on velocity tuning in single neurons [24] and complex kinematic models [25], did not contain the robust rotational patterns they observed empirically, and therefore are weak descriptive models [23].

However, it has been shown that when fitting neural activity to kinematic variables, decoding of movement intention can be improved by including variable time lags between single neuron activity and kinematics (neuron-kinematic latency, [24,26–28]) and these offsets are highly variable (SD: 70 ms; re-digitized data, Moran & Schwartz [24], their Fig 13A). Yet, these offsets were not included in the comparison to representational models made by Churchland et al. [22]. Furthermore, given that representational models of single neuron tuning have been widely implemented in both an experimental and clinical setting, such as in the development of neural prosthetics, it is not clear how those results can be interpreted under the dynamical systems framework.

To clarify this, we first investigated whether or not jPCA would reveal rotational dynamics in a velocity-based model for center-out reaching in which neuron-kinematic latencies were built into single neuron activity. We found that jPCA alone revealed rotational structure in both the representational model and the dynamical model, but that implementing a novel covariance-matched permutation test (CMPT) readily distinguished between these two, showing that variable neuron-kinematic latency did not uniquely produce rotational structure due to the condition structure. Secondly, we show that movement intention could be decoded from a recurrent neural network (RNN) trained to complete the same task using representational methods, such as the population vector, even though the preferred directions of single neurons were highly unstable, suggesting that high levels of decoding performance using representational models do not necessarily inform the mechanistic operation of the underlying circuit. Importantly, both simulated RNN data and real data collected in PMd/M1 of macaque monkeys show similar and significant rotations under the CMPT, providing further support for the dynamical systems view. Furthermore, repeating the CMPT on subsets of the PMd/M1 data showed clear minima in number of neurons and conditions required to draw statistical conclusions, cautioning the use of such analysis methods on low numbers of conditions or neurons, and thus informing the design of future experiments.

## Results / Discussion

### Incorporating variable neuron-kinematic latencies into the representational model

**Fig 1:**
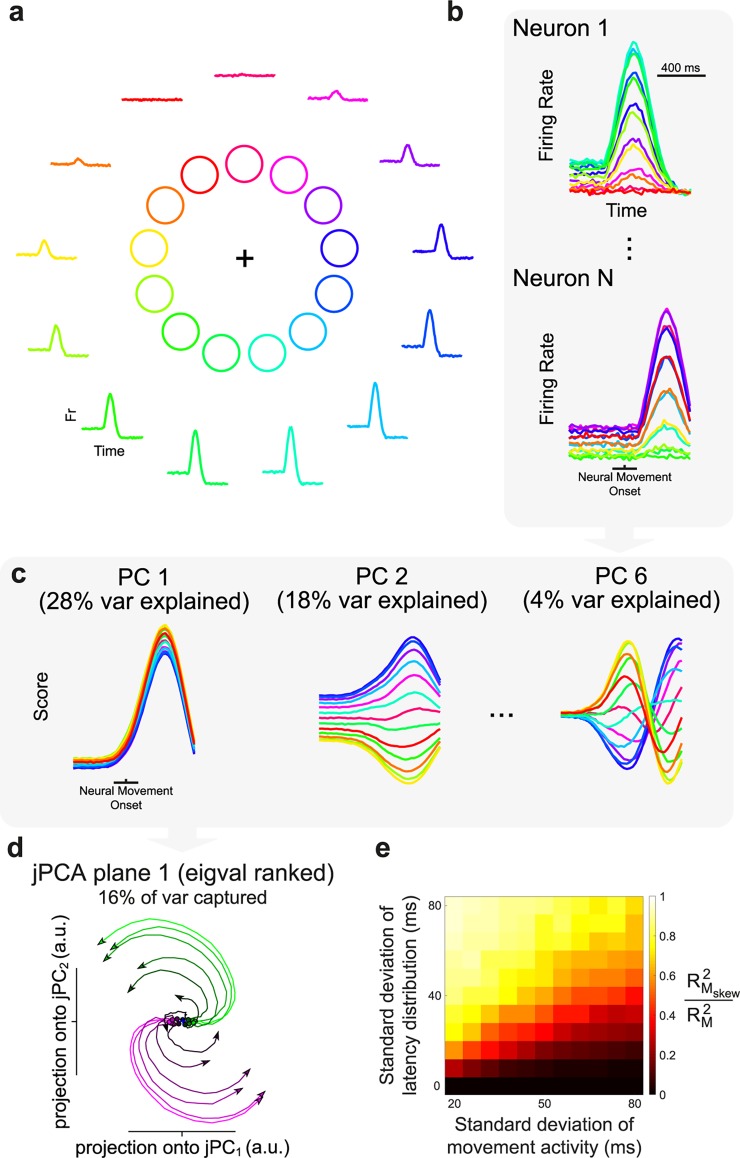
Simulation of a velocity-tuning based model with variable neuron-kinematic latencies. (**a)** Task design of a 13-direction center-out reaching task. The firing of a simulated neuron is plotted around the reach directions. (**b**) Two example neurons with differing latencies. (**c)** Principal components (PCs) for a simulated population of 200 neurons (latency SD: 72 ms, movement SD: 56 ms). (**d)** Exemplar jPCA plane for the first 6 PCs of the simulated population from 0 ms before to 200 ms after neural movement onset (analysis was computed on entire movement). Individual conditions are colored based on their activity at neural movement onset in the first jPC. (**e)** Proportion of change in neural trajectory explained by rotational dynamics (in all jPCA planes) for various latency offsets and movement durations. A value of 1 indicates that rotational dynamics completely explain the transformation between each time point and its temporal derivative.

Velocity-based models without variable neuron-kinematic latencies were shown to exhibit little to no rotational structure [22]. To investigate how variable neuron-kinematic latencies may affect rotational structure, we simulated 200 cosine-tuned motor cortex neurons in a standard 13-direction center-out reaching task with variable neuron-kinematic latencies (Fig 1A; Methods) [13]. The simulation was based on the assumption of bell-shaped velocity profiles (Fig 1B). For activity with a movement duration of 300–400 ms and a latency distribution with a standard deviation (SD) of 72 ms, we found that the first principal component (PC) of our population of simulated neurons resembled a condition-independent representation of the individual neuron profile, while the second PC resembled a condition-dependent representation (Fig 1C). Interestingly, all higher order PCs resembled a sequence of harmonic Fourier bases. In general, it is well known that time-shifted versions of identical signals preferentially produce PCs very similar to a Fourier series (S1A and S1B Fig) as a result of sinusoidal eigenvectors of increasing frequency. This feature introduces a potential confound, since the higher-order PCs show patterns of activity that are not present in any individual neurons. Furthermore, these PCs produce rotational ‘horseshoe’ patterns when plotted in a plane (S1C Fig) [29], revealing how rotations can emerge from signals that are not present in any individual neuron (for an example of false interpretations made from application of PCA, see this well-known example from genetics research [30,31]).

In order to test the presence of rotational structure at the population level, we implemented the same analysis developed by Churchland et al. [22], termed jPCA (Methods). jPCA is a method for finding linear combinations of principal components that capture rotational structure in a population of neurons. In essence, jPCA finds low-dimensional planes in which neural activity follows a predictable rotational trajectory from time point to time point (analogous to a circular flow-field). We found that the introduction of the above-mentioned variable neuron-kinematic latencies were sufficient to produce rotational dynamics (Fig 1D) when explored with jPCA, unlike the representational model results of Churchland et al. [22], who found only weak rotations. The level of rotational dynamics observed here is similar to empirically recorded PMd/M1 data in terms of visualization of the jPCA planes, amount of variance explained per plane (30% in the first two planes, 16% in the first plane), rotational goodness-of-fit ratio (RGR) between $R_{M_{skew}}^{2}$ and $R_{M}^{2}$ (0.79 in the first three planes; Methods), which provides a measure of how much variance can be explained by purely rotational dynamics, and how circular the rotation (0.72, where 1 is purely circular, computed as the average dot product of angle between *x* and $\dot{x}$, and *π*/2; Methods).

To characterize more generally how rotational structure arises with the addition of variable lags, we varied the duration of movement period activity (expressed as the SD of normally distributed movement activity; Methods) and the SD of the latency distribution systematically in repeated simulations (Fig 1E). Interestingly, when the SD of the latency distribution exceeded the SD of the movement activity, the level of underlying rotational structure increased rapidly. Therefore, our results show that the application of jPCA alone on a population where neuron-kinematic latency is more variable than the duration of movement leads to rotational dynamics.

### Disrupting the underlying condition structure–covariance-matched permutation test

Based on the above results, it is clear that jPCA alone is not sufficient to distinguish between a representational model with lags and the dynamical model proposed by Churchland et al. [22]. While Churchland et al. [22] performed extensive shuffling controls to test the possibility that rotations emerge purely as a consequence of high-dimensional data, their controls do not differentiate between the above cases. Therefore, we developed a covariance-matched permutation test (CMPT) to differentiate these models. The objective of our test was to determine if the underlying condition structure, i.e., whether or not shuffling the neural data between different task conditions independently for each neuron, uniquely determined the rotational structure as is predicted by the dynamical model.

**Fig 2:**
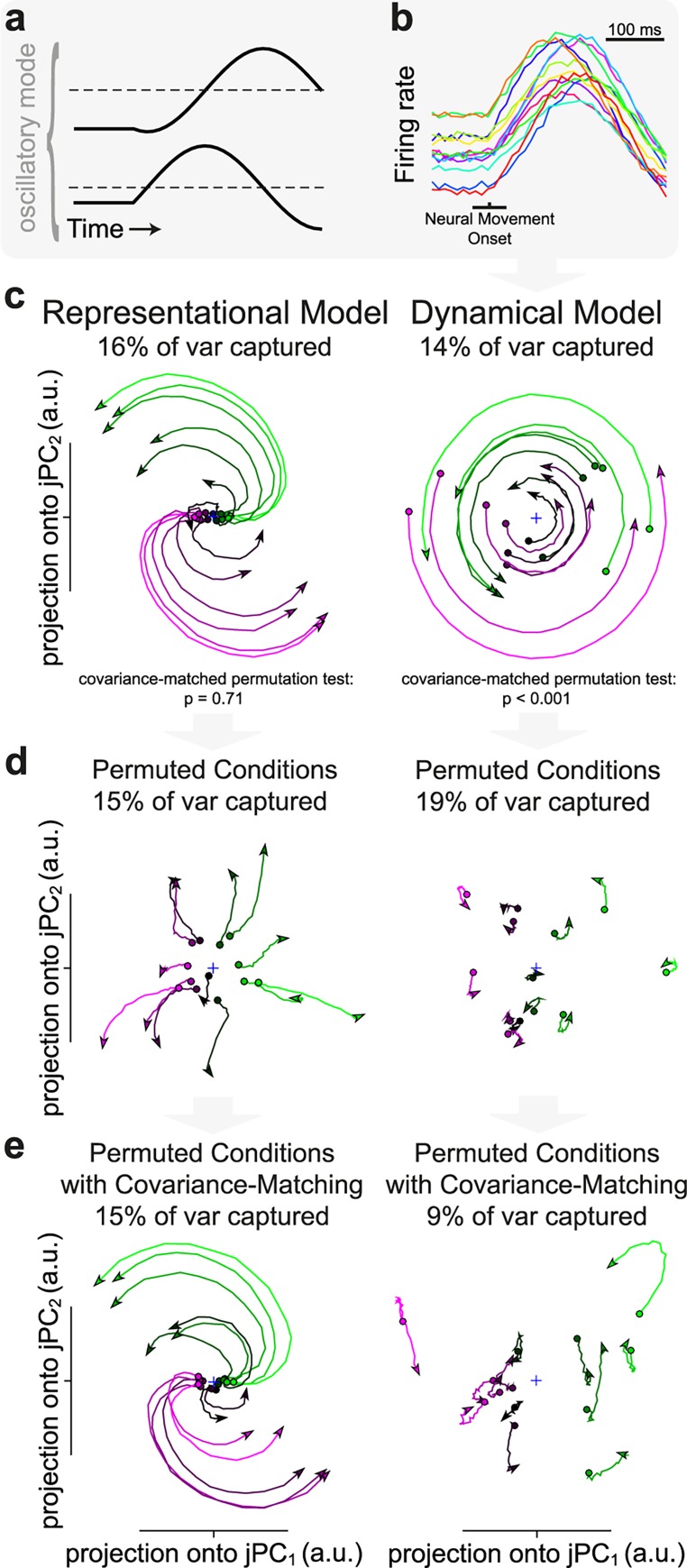
Comparing rotational structure between the representational and the dynamical models. (**a**) One of the two oscillatory modes (2.8 Hz) used to generate the simulated muscle activity of all conditions (2.8 Hz and 0.3 Hz). (**b**) Firing rate of an example neuron of the dynamical model for all 13 conditions. Each neuron is generated from a random combination of the two underlying oscillatory modes and offset for each condition. (**c**) Rotational dynamics in the first jPCA plane for the observed data. *p*-value shows results of CMPT for the representational and dynamical models evaluated by the rotational goodness-of-fit ratio (RGR: $R_{M_{skew}}^{2}/R_{M}^{2}$). (**d**) Same as **c**, but for permuted data without covariance matching. (**e**) Same as **c**, but for covariance-matched data. Data is plotted for 200 ms regardless of time period used to generate statistics. Colors are based on the preparatory activity in the first jPC.

To provide intuition about the rationale of the test, consider the dynamical model proposed by Churchland et al. [22]. They observed that muscle activity during reaching could be fit extremely well (correlation coefficients ≥ 0.97) by a summation of two sinusoidal oscillators, each with fixed frequency, but whose phase, amplitude, and constant offset varied from condition to condition (Methods). They proposed that these oscillators underlie the neural population activity during movement, providing a basis set from which the muscle activity can be generated, while the preparatory activity sets the phase and amplitude of these rotations. Since the phase and amplitude of these rotations are unique to each condition and defined jointly across the entire neural population, disrupting the condition structure should eliminate rotational structure. In Fig 2A we show one of two example oscillators (2.8 Hz), which consisted of a pair of leading and lagging sinusoids. To simulate neurons in the model, we randomly combined the oscillatory signals and offset, where each condition had a different phase, amplitude, and offset (Fig 2B; Methods; see Churchland & Cunningham [23], their Fig 2, for another illustration).

After applying jPCA, Fig 2C shows that strong rotations exist at the population level for both the representational model (same as Fig 1D) and the dynamical model (28% variance explained in first two planes, 14% in the first plane, 0.97 RGR, 0.98 circularity). In order to test if the underlying condition structure was uniquely responsible for the observed rotations, the CMPT consisted of reassigning task conditions within individual neurons while maintaining the overall covariance matrix between all neurons to a reasonable threshold (95% similarity; Methods). This method disrupts the underlying relationship between neurons and conditions, but not other measures, such as average rate per neuron, relationship between neurons in the population (covariance), and each neuron’s contribution to each PC, since the results of PCA are dependent on covariance. If rotations are disrupted as a result of our control, the underlying relationship between neurons and conditions is uniquely essential to the emergence of rotations. On the other hand, intact rotations indicate that many possible condition assignments produce similar rotational patterns, at odds with the findings of Churchland et al. [22] in PMd/M1 data.

Initially randomly permuting conditions without covariance matching destroyed rotational structure in both the representational and dynamical models (Fig 2D). However, after repeating the CMPT procedure (1000 repetitions) and comparing the RGRs between the observed and permuted data sets to generate a *p*-value (Methods), we found that the rotational structure found in Fig 2C was restored after covariance matching in the representational model (Fig 2E, *p* = 0.71), but not for the dynamical model (*p* < 0.001, Fig 2E). As a further measure of statistical power, the effect size of rotations in the dynamical model was quite high (effect size: 3.2; Eq 4 in Methods).

In the representational model, permuting disrupts the condition structure, but not the lag relationships, since no data is exchanged between neurons. Once the overall neuron-to-neuron relationship is restored after covariance matching, the rotations are restored as well, even though the condition structure is still disrupted, showing that rotational structure in the representational model does not emerge because of a unique condition structure, as it does in the dynamical model. Repeating the same analysis on additional simulations where neurons were permitted to achieve both positive and negative firing rates (b_n,c_ = cos[θ_c_ – θ_n_] in Eq 1), or when the magnitude of kinematic tuning per neuron varied randomly, did not alter this result (*p* = 0.92 and *p* = 0.22, respectively). Furthermore, the CMPT did not simply ‘unshuffle’ the data, as there was no significant correlation between the RGR of a given permutation and how similar the condition assignment in that permutation was to the original condition assignment in the observed data (Methods; representational model: r = 0.03, *p* = 0.30; dynamical model: r = 0.03, *p* = 0.49).

It remains an open question whether or not the CMPT can also distinguish rotations arising in a dynamical model from those generated by a complex-kinematic model with varying neuron-kinematic latencies, in which neurons are not only sensitive to velocity, but also to position, acceleration, and occasionally jerk [25]. Therefore, we simulated a population of neurons identically to the representational model (Methods; Fig 1D), but further implemented sensitivity to these additional kinematic parameters with the same weights as Churchland et al. [22] (S2A Fig; assuming a reach radius of 20 cm). While complex-kinematic model simulations with no varying neuron-kinematic latencies only produce weak rotations (see Churchland et al. [22], their Fig 4), the inclusion of lags generated rotational structure (S2B Fig; RGR: 0.89, circularity: 0.82). However, similar to the representational model, these rotations were not significant under the CMPT (*p* = 0.09), further emphasizing the power of the CMPT in identifying rotations that are uniquely dependent on the underlying condition structure.

Repeating the CMPT on the representational model for all parameter combinations in Fig 1E revealed that these data generally had no significant rotational structure (*p*-values above 0.05, 100 permutations). Occasionally, *p*-values below 0.05 occurred, but the magnitude of these effects were extremely small and completely disappeared for stricter implementations of the CMPT (similarity 99%), a modification that had no impact on the dynamical model. Taken together, these findings suggest that a broad variety of simulated populations of classically cosine-tuned neurons can exhibit reasonably strong rotational dynamics when explored using jPCA, but that proper controls disrupting the underlying relationship between conditions while conserving other features can distinguish these rotations from those proposed by the dynamical model.

### Hallmarks of representational tuning and rotational structure in a recurrent neural network model

**Fig 3:**
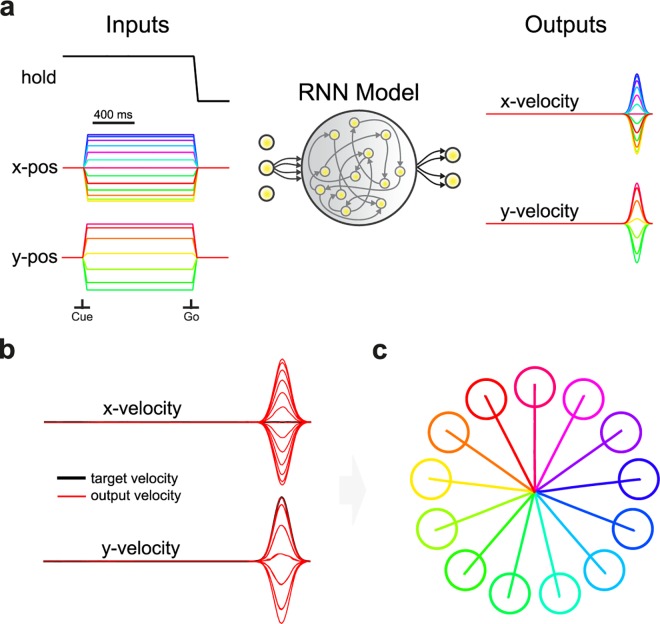
Schematic of recurrent neural network performing center-out reaching. (**a**) Schematic of RNN, with input layer, hidden layer, and output layer. The three inputs were a condition-independent hold signal that was released at the go cue and two inputs representing the target angle. The two outputs were a linear combination of the internal neurons and read out velocity in the x and y direction. All weights were modified during training. The network received no feedback from the output layer. (**b**) Output velocity profiles produced by the RNN compared with target velocity used in training. The normalized error was less than 0.1%. (**c**) Simulated kinematics produced by integrating the velocity profiles over time, with corresponding targets for illustration.

Given that representational tuning models have been used extensively to characterize motor cortex activity, how can findings of robust single neuron tuning be reconciled with a dynamical model of movement generation? To address this question, we implemented a simple recurrent neural network (RNN), operating as a dynamical system, from which the velocity profiles required to complete the previously described center-out reaching task can be read out (Fig 3A; Methods). Recent studies have augmented the original findings of Churchland et al. (2012) by generating biologically plausible RNNs that seek to produce complex activity patterns [20,32,33] and using cortical circuit models to explain population activity [34].

In accordance with recent work [20,32], we constructed two time-varying inputs representing the location of the target in 2-D space, and one input representing a hold signal that is released at the go cue. As in the representational model, we generated a network with 200 internal neurons (Methods). The outputs of the network were the x- and y-velocity profiles of the reach. After training, the RNN was able to withhold movement for the entire delay period and execute accurate velocity profiles with a normalized error of less than 0.1% (Fig 3B). Integrating the decoded velocity over time produced the desired kinematics for each reach direction (Fig 3C). A benefit of such a framework is that preparatory activity cancels out at the level of the output signal (null-space), as output must be suppressed during planning to avoid premature movement, a quality observed empirically between PMd/M1 and muscles [35].

**Fig 4:**
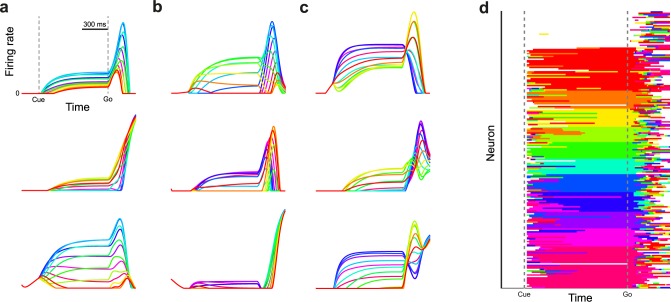
Tuning properties of RNN neurons. (**a**) Three example units for which the pattern of directional tuning remained highly correlated between the delay period and movement. (**b**) Same as **a**, but for example units that have delay tuning that is not correlated with movement activity. (**c**) Same as **a**, but for example units that invert their tuning between delay and movement. (**d**) Preferred reach direction (highest firing) of all 200 units, sorted by preferred direction at go cue. If there was no firing rate difference (< 1e-4) between the preferred direction and non-preferred direction, units were deemed un-tuned and are marked in white. Firing rates are displayed from 0 to the maximum firing rate of each neuron.

Fig 4A shows the responses of three example neurons that showed very similar tuning patterns during the delay and movement. Fig 4B shows examples in which the delay tuning was unrelated to movement tuning, and Fig 4C shows examples where the tuning preference flipped at various times during the movement. The overall diversity of tuning is similar to motor cortex neurons presented in Churchland et al. [22] and Sussillo et al. [20].

Fig 4D shows the preferred reach direction (highest firing) of all 200 simulated neurons over time. Preferred directions remained relatively stable during the late delay period, but shortly after the go cue the preferred directions changed rapidly [36]. In this framework the neurons themselves are not explicitly tuned for any given reach direction and are expected to vacillate when the network is released, a property observed previously in a feed-forward network with state feedback (Lillicrap & Scott [7], their Fig 2F).

**Fig 5:**
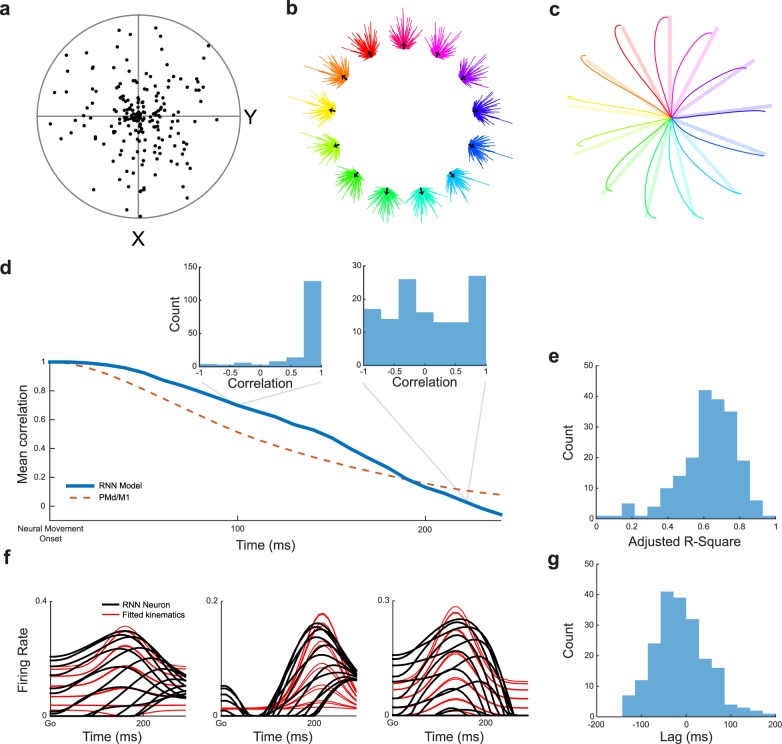
Representational tuning in an RNN for center-out reaching. (**a**) Preferred movement direction in Cartesian space of all units, corresponding to the magnitude of *b*_*i*,2_ and *b*_*i*,3_ in Eq 9. (**b**) Summary of contribution vectors of all individual neurons (one vector each) over the entire movement, with black population vector showing the overall predicted movement direction. (**c**) Integrating the population vectors in panel **b** over time traces out a predicted trajectory (solid) that largely matches the actual trajectory (translucent). (**d**) Mean correlation between condition tuning order at neural movement onset compared to later time points during movement (in steps of 10 ms) for the RNN model and an example PMd/M1 data set presented in Churchland et al. [22]. Insets show full correlation histograms for two time points. (**e**) Adjusted R-Square obtained by regressing the activity of each neuron (from the go cue to the end of movement 300 ms after go) on a representational cosine model of velocity tuning (Methods). (**f**) Movement activity of three example neurons and the corresponding velocity based regression fits. The overall fit performance to these units is high (Adjusted R-Square above 0.8), but the regression fails to capture the multiphasic and varied nature of the underlying signal. (**g**) Time lag between neural activity and velocity, per neuron, obtained from the velocity tuning regression in panel **e**, showing a large range of values.

One of the most iconic movement prediction techniques is the population vector, which has been used extensively to decode intended movement direction and instantaneous velocity using knowledge about the preferred direction of all neurons in a population [37,38]. Fig 5A shows the preferred directions of our model neurons (Methods), which were distributed throughout the Cartesian space. Fig 5B shows contribution vectors of all individual neurons over the entire movement of each condition, revealing a remarkably good prediction of movement direction (mirroring results of Georgopoulos et al. [38], their Fig 1). Lastly, Fig 5C shows the result of integrating all population vectors over the course of movement, producing predicted trajectories that well match the desired trajectories (mirroring results of Georgopoulos et al. [38], their Fig 5). In addition, tuning curves of individual RNN neurons visually resembled those observed empirically (S3 Fig). Together, these results reveal that readouts based on the assumption of “preferred direction” can accurately reproduce intended trajectories even when consistent individual neuron tuning was neither included nor observed in the model, a feature of the population vector that has been mathematically outlined by Sanger [39].

As we saw in Fig 4D, preferred direction seemed to fluctuate throughout movement. By correlating the average firing of each neuron for each condition between neural movement onset and later time points during the movement, we can track the stability of tuning over time. The more time has elapsed since neural movement onset, the lower the correlation between delay tuning and movement tuning (Fig 5D; mirroring results of Churchland et al. [40], their Fig 4), both in the model and in example data from PMd/M1 (data from [22], Monkey N). Furthermore, the distribution of correlation coefficients across the population is not bimodal, a finding that would be expected if one subpopulation of neurons was positively correlated over time and one subpopulation inversely tuned during movement.

Based on the above finding that preferred directions are highly variable during movement, how can it be that representational tuning models explain large amounts of variance in firing rate in empirical studies [24]? Interestingly, regressing the movement activity of each neuron on a full model of velocity tuning (Methods) produces fits very similar to empirical data (Fig 5E, mean Adjusted R-Square: 0.63, mirroring results in Moran and Schwartz [24], their Fig 12A and 12B). However, the actual model fits do not well capture the dynamic properties of the individual units (Fig 5F), such as the changes in preferred direction that occur over the course of the movement or non-linear changes such as when neurons cease firing (0 Hz). Importantly, the optimal neuron-kinematic offsets obtained in the regression cover a range of values, very similar to those observed previously (Fig 5G, mirroring results in Moran and Schwartz [24], their Fig 13A and 13B), providing a potential explanation of how variable neuron-kinematic latencies can improve the performance of representational tuning models even when fixed offsets between neurons is not a property of the underlying circuit.

**Fig 6:**
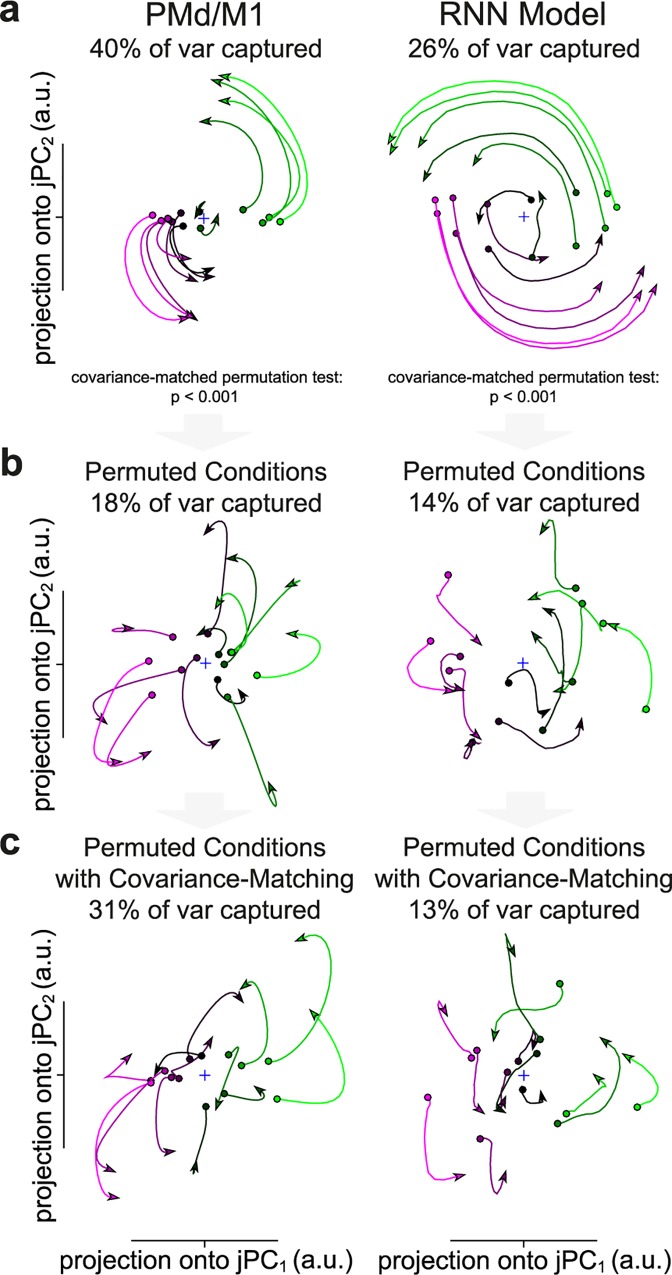
Significant rotational structure in PMd/M1 data and RNN model. Comparison of rotational dynamics for (**a**) observed, (**b**) permuted without covariance matching, and (**c**) covariance-matched data in the first jPCA plane. *p*-values in **a** are from the CMPT for the rotational goodness-of-fit ratio (RGR: $R_{M_{skew}}^{2}/R_{M}^{2}$) in all jPCA planes. Conditions and neurons were randomly down-sampled in the PMd/M1 data to match the RNN model. Data is plotted for 200 ms regardless of time period used to generate statistics. Colors are based on the preparatory activity in the first jPC.

Yet, it remains unclear if significant rotational structure underlies the activity of our RNN. Therefore, we repeated the jPCA analysis and CMPT with both example data from PMd/M1 and our RNN model. As seen in Fig 6, the PMd/M1 data contained robust rotational structure explaining 56% of the variance in the first two planes (40% in the first plane), an RGR of 0.77 over all jPCA planes, a circularity of 0.63, and the rotational structure was highly significant (*p* < 0.001, CMPT with 1000 repetitions). Importantly, the RNN model also produced robust rotations, explaining 54% of the variance in the first two planes (26% in the first plane), an RGR of 0.74 over all jPCA planes, a circularity of 0.73, and the rotational structure was highly significant (*p* < 0.001, CMPT with 1000 repetitions). In both cases the effect size was also very large, 4.1 and 3.7 for the PMd/M1 data and RNN model, respectively. In addition, similarly to the representational and dynamical models, the CMPT did not simply ‘unshuffle’ the condition assignment, as the correlations between the RGR of each permutation and the similarity in condition assignment to the observed data was not significant for the PMd/M1 data (r = 0.06, *p* = 0.06) or the RNN model (r = -0.002, *p* = 0.94).

**Fig 7:**
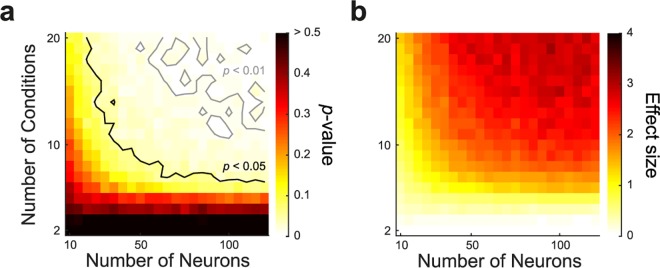
Number of neurons and conditions required for statistically significant rotations. The CMPT was carried out (500 repetitions) for many subsets of example PMd/M1 data including from 10–120 neurons and 2–20 conditions. (**a**) Map of *p*-values for the rotational goodness-of-fit ratio (RGR: $R_{M_{skew}}^{2}/R_{M}^{2}$). (**b**) Map of effect size (difference between observed RGR and mean of permuted distribution, divided by the SD of the permuted distribution). For every permutation, random neurons and conditions were drawn from the example set. Contours show the 0.05 and 0.01 significance levels.

Although significant rotational structure was found in the PMd/M1 data, it is unclear how many recorded neurons and conditions are necessary for jPCA to reveal this result. Therefore, we repeated the CMPT on many subsets of the PMd/M1 data by randomly sampling conditions and neurons to determine how many neurons or conditions might be required to produce statistically significant rotations (Fig 7). This analysis revealed that our test was able to identify clear minima in number of neurons and conditions that are necessary to achieve significance, in general more than 30 neurons and more than 8 conditions, a finding that may guide the design of future experiments and encourages skepticism of experiments with small numbers of neurons or conditions.

It is important to note that the CMPT may not necessarily distinguish between all possible models, as there exist cases of the dynamical model for which our test would find no significant rotational structure. For example, if the required oscillator phases required to fit muscle activity were identical between all conditions, while rotational structure would be found using jPCA, our test would find these rotations to be non-significant. Therefore, we do not propose the CMPT as a singular test of rotational structure to accompany jPCA, but rather as an additional control.

We posit that future studies should seek to explain single neuron characteristics as a function of population or circuit activity rather than imbue single neurons with complex tuning characteristics [9,10]. Furthermore, RNNs provide an ideal medium for more detailed study, as the ground truth of synaptic connectivity, plasticity, noise, trial-to-trial variability, and responses to unexpected perturbations are known and can be manipulated directly. However, “exploring an artificial model universe comes with its own risk” [41] and proper models must resist the temptation of explaining purely idiosyncratic properties, but rather those that are able to explain large amounts of variance in electrophysiological data. Our results also emphasize that explaining a large amount of variance in neural data in and of itself does not necessary lead to mechanistic insight [42], as the observation of rotational structure arose under multiple models, and future work is needed to determine the biological circuit mechanism underlying population level rotational structure.

Fundamentally, as representational [43,44] and dynamical [20,32,45] models become more complex in their implementations, their ability to explain empirical data becomes more striking and convincing. Ultimately, what will signify the usefulness of either framework will be their utility in generating testable hypotheses of how the brain executes complex behavior in basic research contexts, and in developing new solutions in applied research contexts. In terms of application, the representational view has been indispensable in developing neural prosthetics for paralyzed patients [46–48], but this trend may be changing as prosthetic algorithms are augmented by the inclusion of dynamical systems into their underlying framework [49,50].

## Methods

### Representational model

Preparatory and movement activity were simulated for a population of 200 neurons in a 13-direction center-out reaching task. Neurons were cosine-tuned for velocity during both preparation and movement with respect to their randomly assigned (uniform) preferred direction. The average firing rate, *f*_*n*,*c*_, of a given simulated neuron, *n*, for a particular reach condition, *c*, at time *t* is given by,

$$f_{n,c}\left(t,\tau_{n},\sigma \right)=\{\begin{array}{ll} \begin{array}{l} b_{n,c}e^{-\frac{{\left(t-\tau_{n}-\mu_{0}\right)}^{2}}{2\sigma^{2}}}+\varepsilon ,\;t\geq \tau_{n}\\ \varphi b_{n,c}+\varepsilon ,\;t<\tau_{n} \end{array} & ,\;b_{n,c}=\frac{1+cos\left[\theta_{c}-\theta_{n}\right]}{2} \end{array}$$ (1)

where *τ*_*n*_ is the neural response latency (normally distributed) of each neuron, *σ* is the duration parameter of the movement activity, which never differed between neurons of the same simulation, *b*_*n*,*c*_ is the gain factor for each neuron and condition, *θ*_*c*_ is the angle of the reach target in condition *c*, *θ*_*n*_ is the preferred reach angle of neuron *n*, *φ* a constant which determines the magnitude of preparatory activity, *μ*_0_ a constant and given by $\mu_{0}=\sigma \sqrt{-2ln\varphi }$, and *ε* is random noise drawn from a normal distribution.

For all analyses, *φ* was fixed at 0.2, i.e., preparatory activity was always one fifth of the maximum movement activity for that condition; however, our results do not depend on this factor. The distribution of latency factors, *τ*_*n*_, and the movement duration parameter, *σ*, were varied systematically to produce the results in Fig 1E. For visualization purposes we chose the noise distribution, *ε*, to have an SD of 0.01 for all analyses. However, this value did not greatly affect the outcome. We found it necessary to increase the noise more than 300 times to eliminate all structure.

### Rotational dynamics

jPCA is a method for finding linear combinations of principal components that capture rotational structure in data (Churchland et al., 2012). The method is based on finding a transformation between a neural system at each time point and its temporal derivative, using the following steps. First, the average firing rate of many neurons is extracted and aligned to the execution of a movement, starting whenever the neural activity begins rapidly changing preceding movement onset, termed neural movement onset (typically 100–200 ms before overt movement). Next, each neuron is normalized and reduced, using standard principal component analysis, to a set of principal components, *X*_*red*_, of size *d* × *ct*, in which the *d* largest components are retained, and *c* is the number of conditions and *t* is the number of time points selected. Then, via linear regression, the unconstrained matrix *M* and the skew-symmetric matrix *M*_*skew*_ (where *M*_*skew*_ = −*M*_*skew*_^*T*^) can be found to satisfy ${\dot{X}}_{red}=MX_{red}$ and ${\dot{X}}_{red}=M_{skew}X_{red}$, where ${\dot{X}}_{red}$ is the difference in adjacent time points of *X*_*red*_ (temporal derivative). The jPCA planes are then constructed from the eigenvectors of *M*_*skew*_, with the added constraint that the net rotation in each plane is anticlockwise.

In order to avoid finding spurious rotations, only the first 6 PCA dimensions explaining the most variance were fed into the jPCA algorithm (sampled in steps of 10 ms). For the representational model, the data fed into jPCA began at neural movement onset, which was defined as the time when the average signal exceeded 10% of the difference between preparatory activity and maximum activity, and ended when the average activity fell below this level. Given the variable lags between neurons, it was necessary to define the above procedure for determining neural movement onset, which is similar to the one performed by Churchland et al. [22]. For the dynamical model and RNN model, neural movement onset was simply defined as the time of the go cue, and the entire movement (300 ms) was used. On the other hand, for the example PMd/M1 data (presented as Monkey N in Churchland et al. [22]) the analysis was replicated as done in Churchland et al. [22], from -50 to 150 ms relative to neural movement onset, a time-course specifically chosen to avoid sensory feedback not present in our simulation. jPCA was performed using a freely available toolbox (http://churchlandlab.neuroscience.columbia.edu/links.html).

### Dynamical model

The dynamical model is based on the finding that muscle activity during reaching can be well explained by a summation of the lagging components of two oscillatory modes, each with a fixed frequency, but with varying phase, amplitude, and offset for each movement condition [22,23]. Following Churchland et al. [22], we simulated for each condition *c* = *1*,…,*13* an offset *o*_*c*_, and the two complex oscillations (*k* = *1*,*2*)

$$F_{c,k}=a_{c,k}\;e^{i\left(2\pi f_{k}\;t-\theta_{c,k}\right)}$$ (2)

for the two underlying frequency modes *f*_1_ = 2.8 *Hz* and *f*_2_ = 0.3 *Hz* (however, the specific frequencies used did not alter the results). Phases, *θ*_*c*,*k*_, amplitudes, *a*_*c*,*k*_, and offset, *o*_*c*_, were randomly drawn for each condition to match both the variance explained per plane and the similarity between conditions in the representational model (phase drawn from uniform distribution, range: 0 to *π*/2; amplitude drawn from uniform distribution, range: -1.5 to -2.5; offset drawn from uniform distribution, range: -4.5 to -5.5). For simplicity, we did not implement the windowed gamma functions used in Churchland et al. [22], as these only increase the realism of neural responses and do not contribute to the main result.

To generate simulated neurons in the dynamical model (N = 200), the activity *r*_*n*,*c*_(*t*) of each neuron *n* ∈ {1,…,*N*} and condition c was generated as a neuron-specific combination of the condition-specific oscillations and offset (*F*_*c*,1_, *F*_*c*,2_, *o*_*c*_)

$$r_{n,c}\left(t\right)=Re\left(w_{n,1}\;F_{c,1}\left(t\right)+w_{n,2}\;F_{c,2}\left(t\right)\right)+s_{n}o_{c}+\varepsilon_{n,c}\left(t\right)$$ (3)

with the real and imaginary components of the complex coefficients, ***w***_*n*,1_ and ***w***_*n*,2_, and the offset coefficient *s*_*n*_ drawn from a standard normal distribution (zero mean, unit variance). As described above, each neuron had a unique set of 5 weights that were used for all conditions, and the small amount of normally distributed noise *ε*_*n*,*c*_(*t*) that was matched to the representational model. Preparatory activity (*r*_*n*,*c*_(*t*) for t<0) was generated by simply extending the first data point, i.e., *r*_*n*,*c*_(0) including noise, for 100 ms back in time.

### Covariance-matched permutation test for rotational dynamics

In order to test if the rotational dynamics found in neural population data depended on the underlying condition structure, we developed a covariance-matched permutation test to disrupt the condition-wise relationships while sparing other features of the data. In this iterative procedure the entire time-course of each condition, for each neuron separately, was first randomly reassigned to another condition. Then, individual pairs of conditions were randomly exchanged (within, but not between random neurons) and the similarity of the covariance of all neurons was compared to the observed data for the time period of interest (i.e. the time period analyzed using jPCA). Covariance between neurons was calculated from the matrix *n* × *ct*, where *n* is the number of neurons, *c* the number of conditions, and *t* the time period of interest. Covariance similarity was calculated as the sum of the squared difference between the observed covariance matrix and the covariance matrix of the permuted data, divided by the variance of the observed covariance matrix. If the similarity was increased by a given permutation, it was accepted, otherwise it was rejected and the process continued. When the covariance similarity between the observed data and the permuted data exceeded 95%, the process was complete (this process generally lasted many thousands of permutations). In this way, no data values were altered. Correspondingly, the average firing rates, total firing rates of all neurons, and the approximate covariance relationship between all neurons were conserved.

To test significance, the permutation procedure was repeated many times (100–1000 repetitions) and the covariance-matched data was fed into jPCA in the same fashion as the observed data. The rotational goodness-of-fit ratio (RGR: $R_{M_{skew}}^{2}/R_{M}^{2}$) over all three jPCA planes (spanning 6 principal components), which provides a measure of how much variance can be explained by rotational dynamics, was evaluated for all permutations. Subsequently, the fraction of repetitions that the above statistic computed from the permuted data exceeded the observed data determined the *p*-value, as is standard procedure for permutation tests.

To measure statistical power, effect size was computed as

$$effect\;size=\frac{RGR_{observed}-{\bar{RGR}}_{permuted}}{\sigma_{RGR_{permuted}}}$$ (4)

similar to Cohen’s *d*.

To test if the results of the CMPT procedure were simply due to ‘unshuffling’ the conditions and restoring the original condition assignment, we checked what percentage of the assignment matrix (*c* × *n*) retained its original condition assignment at the end of the CMPT and correlated this measure with the RGR of the corresponding permutation repetition. Importantly, since even ‘unshuffled’ data would not be guaranteed to be in the same order as in the original data, before correlating we first sorted the rows of the above mentioned assignment matrix by the most common condition in each row (condition 1 most common assigned to row 1, condition 2 most common assigned to row 2, etc.), to achieve the most conservative comparison possible.

### Recurrent neural network

In order to examine a system in which velocity profiles of a 13-direction center-out reaching task could be read out over time, we implemented the dynamical system, $\dot{x}=F(x,u)$, using a standard continuous RNN equation of the form

$$\tau {\dot{x}}_{i}\left(t\right)=-x_{i}+\overset{N}{\underset{k=1}{\sum }}J_{ik}r_{k}\left(t\right)+\overset{I}{\underset{k=1}{\sum }}B_{ik}u_{k}\left(t\right)$$ (5)

where the network has *N* units and *I* inputs, *x* are the activations and *r* the firing rates in the network, which were related to the activations by the rectified hyperbolic tangent function, such that $r=\left\{ \begin{array}{l} \;0,\;x<0\\ tanh(x),\;x\geq 0 \end{array}\right.$. The units in the network interact using the synaptic weight matrix, *J*. The inputs are described by *u* and enter the system by input weights, *B*. The time integration constant of the network is *τ*.

For all simulations N was fixed at 200. The three inputs were a condition-independent hold signal that was released at the go cue, and two inputs representing the target position that corresponded to *sin*(*θ*) and *cos*(*θ*), where *θ* is the angle of the target around the circle. The elements of *B* were initialized to have zero mean (normally distributed values with $SD=1/\sqrt{N}$). The elements of *J* were initialized to have zero mean (normally distributed values with $SD=g/\sqrt{N}$), where the synaptic scaling factor, *g*, was set at 1.5 [51]. We used a fixed time constant of 50 ms for *τ*, with Euler integration every 10 ms.

To produce the two desired velocity profile outputs, which were the x-velocity and the y-velocity of the 13-direction center-out reaching task described previously (bell-shaped velocity profiles lasting 300 ms), we defined a linear readout of the internal network

$$z_{i}\left(c,t\right)=\overset{N}{\underset{k=1}{\sum }}W_{ik}r_{k}\left(c,t\right)$$ (6)

where *z* represents the two velocity readouts (*i* = 1,2) and is a linear combination of the internal firing rates using weight matrix *W*, which was initialized to all zero values.

The input weights, *B*, internal connectivity, *J*, and output weights, *W*, were trained using Hessian Free Optimization [52] using freely available code (https://github.com/sussillo/hfopt-matlab) also utilized in Sussillo et al. [20]. The error function used to optimize the network considered the difference between the output of the linear readout and the desired velocity profiles, *v*,

$$E_{i}\left(c,t\right)=z_{i}\left(c,t\right)-v_{i}\left(c,t\right)$$ (7)

at each time point, *t*, each output dimensions, *i*, and each movement direction, *c*. We report normalized error, which is the sum of the squared error from Eq 7 over all times, dimensions, and conditions, divided by the total variance of the target signal. In addition to the above error signal, we also implemented three regularizations designed to encourage the network to produce biologically-plausible activity (implemented as in Sussillo et al. [20]). The three penalties were a cost on the mean firing rate, the squared-sum of the input and output weights, and a penalty encouraging the network to avoid complex state trajectories (similar to local space contraction [53]). The hyper-parameters used for these regularization were 1e-2, 2e-5, and 5e-5, respectively.

In order to discourage internally-timed responses, the network was trained to produce movements after three varying delays of 600, 800, and 1000 ms. All results used came from the 800 ms delay set and the reaction time (time between go cue and movement onset) of the network was fixed at 100 ms. We opted not to model any feedback, since the goal of the study was to illustrate the main points parsimoniously and without relying on confronting the issue of what kind of feedback is most biologically plausible in such a network.

### Population vector

The population vector decoding technique was performed as described in Georgopoulos et al. [38] and Schwartz et al. [37]. Specifically, the preferred direction of each neuron was determined via linear regression

$$R_{i,c}=b_{i,1}+b_{i,2}sin\;\theta_{c}+b_{i,3}cos\;\theta_{c}$$ (8)

where *R* is the average firing rate of neuron *i* over time from the go cue to the end of movement (300 ms after go) for condition *c*, *b* are constants, and *θ*_*c*_ is the angle of the current target. The preferred direction of each neuron was then defined as

$$C_{i}=\left[\frac{b_{i,2}}{k_{i}},\frac{b_{i,3}}{k_{i}}\right]$$ (9)

where

$$k_{i}=\sqrt{b_{i,2}^{2}+b_{i,3}^{2}}$$ (10)

To make predictions about direction and magnitude of movement [38], the population vector at time *t* during movement was computed using the instantaneous firing rate of all neurons (*R*) and each neuron’s previously determined preferred direction (*C*), such that

$$P\left(t\right)=\overset{N}{\underset{i=1}{\sum }}\left(R_{i}\left(t\right)-b_{i,1}\right)C_{i}$$ (11)

where *N* is the number of neurons. The sum of *P* over all time points during the movement of a given trial then determined the overall predicted direction of movement. Alternatively, *P* could be integrated over time points to trace out a predicted trajectory, as in Fig 5C. Fitting procedure was performed using the Matlab *fit* function using the least-squares method.

### Velocity regression

In order to investigate the presence of representational tuning in the RNN, we regressed the movement period activity of each neuron (starting at the go cue until the end of movement 300 ms after go) on the following model of directional and speed tuning,

$$R\left(t-\tau \right)=a_{1}+\|\vec{V}\left(t\right)\|\left(a_{2}+a_{3}\;sin\left[\theta \right]+a_{4}cos\left[\theta \right]\right)+a_{5}\left(a_{3}\;sin\left[\theta \right]+a_{4}cos\left[\theta \right]\right)$$ (12)

where *R* is instantaneous neural activity, *τ* is the time lag between neural activity and its expression as movement, *a* are constants, *θ* is the direction of the current target, which stays constant during center-out reaches, and $\vec{V}$ is the velocity profile. Fitting procedure and resulting goodness-of-fit statistics were obtained using the Matlab *fit* function using the least-squares method. The final term of the equation was appended in addition to the factors presented in Moran & Schwartz (1999) in order to account for differences in preparatory activity between reach directions, an aspect not utilized in the original experiment when no delay period was present. Tuning during the preparatory period was the same as during movement, scaled by a factor, *a*_5_, which also allowed for inverted tuning during movement.

## Supporting Information

S1 Fig Latency offsets produce derivative-like principal components. (**a**) Firing rates of six simulated neurons (normal distributions with identical SD) over time with random time offsets (drawn from normal distribution). (**b**) The first three principal components of the simulated units. (**c**) The plane formed by the first two principal components, showing a ‘horseshoe’ pattern.
(EPS)

S2 FigSimulation of a complex-kinematic tuning based model with variable neuron-kinematic latencies.(**a**) Four example neurons with differing latencies. (**b-d)** Comparison of rotational dynamics for (**b**) observed, (**c**) permuted without covariance matching, and (**d**) covariance-matched data in the first jPCA plane. *p*-value in **b** are from the CMPT for the rotational goodness-of-fit ratio (RGR: $R_{M_{skew}}^{2}/R_{M}^{2}$) in all jPCA planes. Data is plotted for 200 ms regardless of time period used to generate statistics. Colors are based on the preparatory activity in the first jPC.(EPS)

S3 Fig Tuning curves of RNN neurons during movement. Mean firing rate during the movement epoch of all movement directions for 16 randomly selected RNN neurons.
(EPS)
